# Cytotoxicity studies of an optoacoustic stimulation strategy for the development of laser-based hearing aids

**DOI:** 10.1117/1.JBO.25.6.068002

**Published:** 2020-06-23

**Authors:** Lukas Pillong, Patricia Stahn, Marius Hinsberger, Katharina Sorg, Bernhard Schick, Gentiana I. Wenzel

**Affiliations:** Saarland University, Department of Otorhinolaryngology, Faculty of Medicine, Homburg, Germany

**Keywords:** biocompatibility, 532 nm, laser, optoacoustic stimulation, human fibroblasts, osteoblasts, chondrocytes, hearing impairment

## Abstract

**Significance:** Worldwide, ∼460  million people suffer from disabling hearing impairment. Many of these patients are still not sufficiently supplied with currently available auditory technologies. Optical stimulation of the hearing organ offers a promising alternative for a new generation of auditory prostheses.

**Aim:** To assess the biocompatibility margins of our laser pulse amplitude strategy *in vitro*, we designed a protocol and present the effects on normal human dermal fibroblasts, human chondrocytes, and human osteoblasts.

**Approach:** Laser pulses of 532 nm were applied over 120 s using our laser pulse amplitude modulation strategy. We then assessed cell viability and cytotoxicity through fluorescence staining and quantitative polymerase chain reaction-analysis regarding 84 key player-genes for cytotoxicity and stress response.

**Results:** The first *in vitro* biocompatibility margins for our stimulation parameters applied to cells of the peripheral hearing organ were between 200 and 223 mW ($3348\;{J/\mathrm{cm}}^{2}$). After irradiation with a subphototoxic laser power of 199 mW ($2988\;{J/\mathrm{cm}}^{2}$), only the fibroblasts showed a significant upregulation of GADD45G.

**Conclusion:** Further studies are underway to optimize parameters for the optoacoustic stimulation of the auditory system. Our protocol and results on laser–tissue interactions can be useful for translational laser applications in various other irradiated biological tissues.

## 1 Introduction

Approximately 460 million people worldwide are suffering from disabling hearing impairment. Factors such as growing global population, longer mean life expectancies, and increased exposure to environmental noise contribute to a growing hard of hearing population. Untreated hearing impairment not only leads to a decreased quality of life and social isolation, but also poses an economic burden with annual global costs of ∼750  billion international dollars.^1^

Despite the rapid technological progress and innovations within the field of auditory prostheses, a large number of hard of hearing people are still not sufficiently supplied with the currently available technologies. In addition, many patients who have received conventional hearing aids do not use them regularly or at all. Reasons for this lack of compliance are, for example, insufficient frequency-specific gain, especially in a noisy environment, deficient wearing comfort, feedback and occlusion effects, and recurrent inflammations of the outer ear canal.^2^

Consequently, new stimulation strategies are required to more specifically address the needs of the hard of hearing population. Photons could provide a fast, specific, and contactless energy transfer into vibratory structures of the hearing organ giving rise to a new generation of light-driven hearing aids.

The first local mechanical stimulation of the hearing organ using laser light was reported in 2006 by Fridberger and Ren.^3^ In 2009, Wenzel et al. demonstrated a controlled, contact-free activation of the vibratory structures within the inner ear (Wenzel et al. 2009^4^ and Zhang et al. 2009^5^) and one year later demonstrated the possibility to use this method for the application at different loci, from the tympanic membrane to the inner ear.^6^ However, for frequency specific activation of the hearing organ using optoacoustic stimulation at the eardrum level, a coding strategy based on laser pulse amplitude modulation had to be designed.^7^

To achieve this within the developmental work, we have to take into account the fact that biocompatibility is a fundamental requirement for any medical device. To date, there is only poor knowledge about the effects of 532-nm laser light on human cells and none with the modulation parameters as used in the stimulation strategy described by Stahn et al.^7^ Therefore, in this study, we proposed to establish an *in vitro* cell-culture-based model that would enable us to investigate the effects of our optoacoustic laser amplitude modulation strategy on human cells in parallel to our *in vivo* studies in a mouse model.^8^ These two studies together were planned to define a first biologically safe power range keeping in mind its application for a laser-based hearing aid.

The tympanic membrane is a complex, oval shaped, trilaminar structure, consisting of an outer layer of squamous cell epithelium, a middle layer (lamina propria) formed by fibroblasts and collagen fibers, and an inner layer of mucosal epithelium. The distribution of collagen fibrils in the lamina propria contributes to the elastic properties of the eardrum.^9^ This membrane is anchored and spanned to a fibrocartilage ring along the circumference of the outer ear canal and connected to the bony structure of the malleus at the tympanic side. This architecture enables the tympanic membrane to move in complex vibration modes to transmit energy to the middle and inner ears. The inhomogeneous structure of this tissue with various absorption characteristics at different locations exhibits the potential for complex laser–tissue interactions. For these reasons, we needed to establish a method to assess the cytotoxicity thresholds for our optoacoustic stimulation with 10-ns 532-nm laser pulses in an *in vitro* model using human fibroblasts, chondrocytes, and osteoblasts as three representative cell types for the irradiated tissue.

## 2 Materials and Methods

### 2.1 Cells, Culture Conditions, and Media

In our studies, we used three different adherent human primary cell types to mimic natural conditions as closely as possible: normal human dermal fibroblasts (NHDF), human chondrocytes (HCH), and human osteoblasts (HOB). The cells were cultured in phenol red-free media [fibroblast basal medium 2 (phenol red-free)/chondrocyte basal medium (phenol red-free)/osteoblast basal medium] and the corresponding supplement mix (Promocell, Heidelberg, Germany) to avoid absorption by the media at a wavelength of 532 nm. Adherent cells were seeded out in a 96-well flat-bottom microtiter plate (glass) (Viewplate 96-F, PerkinElmer, Rodgau-Jügesheim, Germany) and covered with 100-μl phenol red-free medium. The cells were incubated at 37°C with 5% ${\mathrm{CO}}_{2}$ for 48 h until the monolayer culture had reached confluence.

### 2.2 Laser Setup

**Fig. 1:**
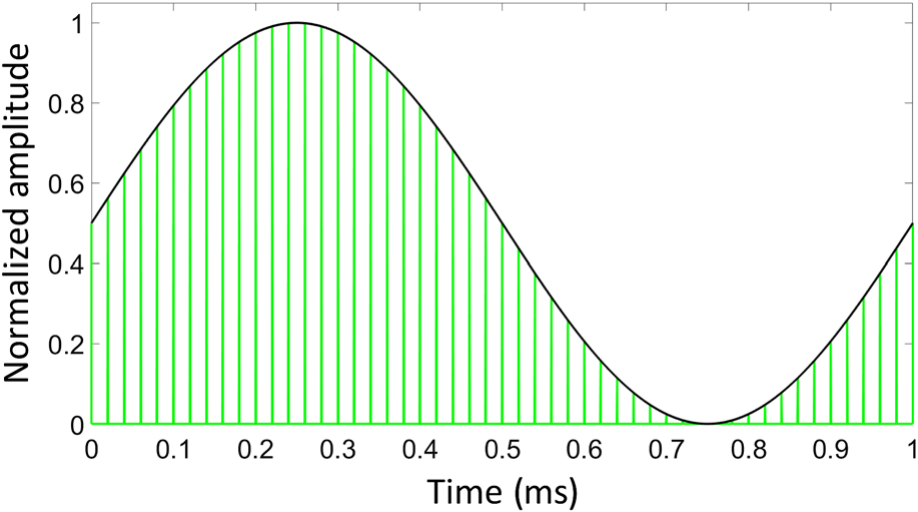
Our laser amplitude modulation strategy with a laser modulation rate of 1 kHz and laser repetition rate of 50 kHz (green vertical lines). (Figure modified from Sorg et al.^8^)

We used a pulsed 532-nm neodymium-doped yttrium orthovanadate laser system (INCA, Xiton Photonics GmbH, Kaiserslautern, Germany). The parameters for the laser amplitude modulation (Fig. 1) were generated as described before^7^ on a personal computer (PC) (Hewlett-Packard Company /HP Inc., Palo Alto, California, USA). The laser system was operated with a predetermined laser pulse rate (LPR) of 50 kHz. We transferred a MATLAB^®^ (R2014a, MathWorks Inc., Natick, Massachusetts, USA) that generated a continuous sinusoid signal to a waveform generator (33500b Waveform Generator, Agilent Technologies, Santa Clara, California, USA) as an arbitrary file via a Virtual Instrument Software Architecture interface. This sinusoid laser signal was sent to the input of the acousto-optic modulator (AOM) (Xiton Photonics GmbH, Kaiserslautern, Germany). The laser pulses were then delivered to the target well using the laser fiber (Ø365  μm) that was connected to the AOM.

**Fig. 2:**
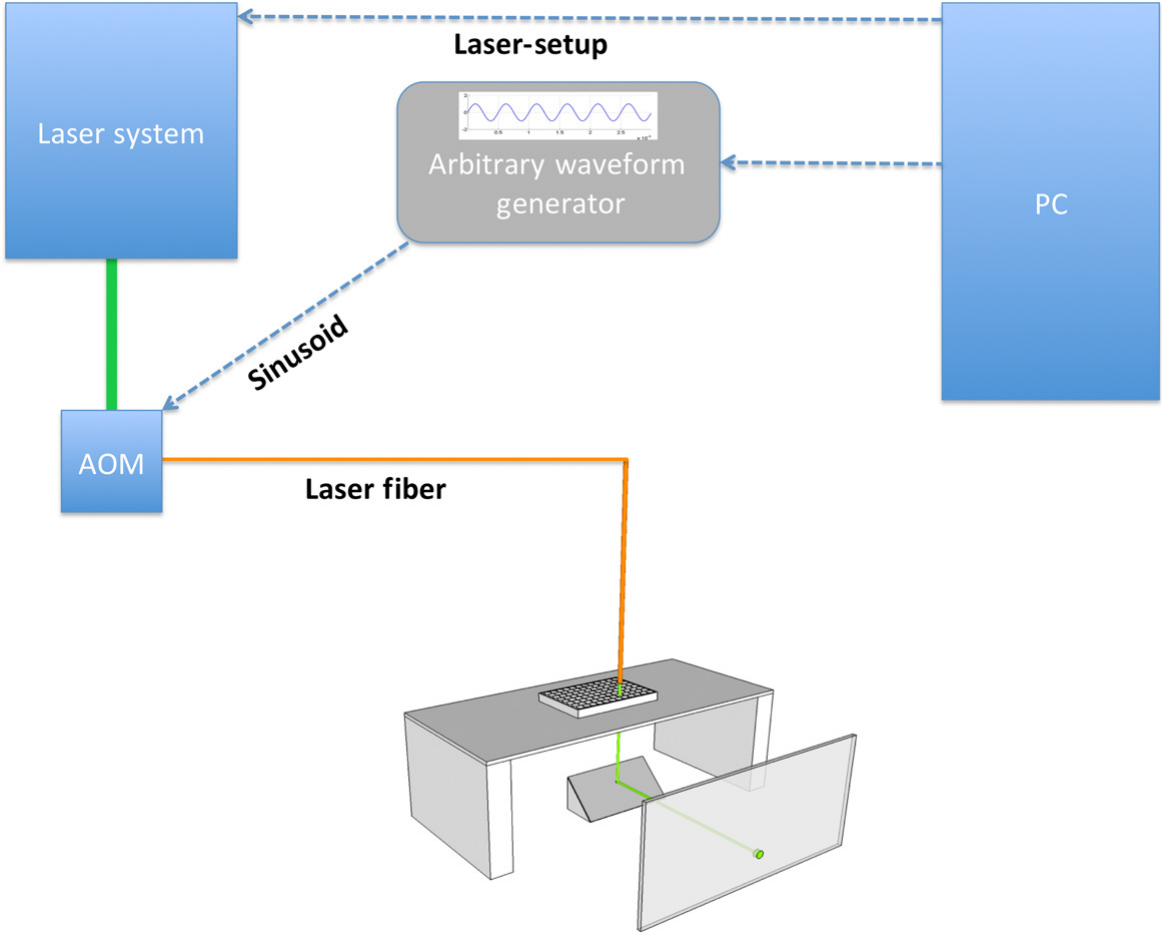
Experimental set up. Laser parameters were defined on a PC via MATLAB^®^. Information concerning the laser power sent to the laser system and the sinusoid signal generated by the MATLAB^®^ was transferred to an arbitrary wave generator and sent to the AOM. A laser fiber connected to the AOM delivered the laser pulses to the target structure.

Before irradiation, the culture medium was removed from two wells: (1) the well that was going to be irradiated and (2) the corresponding untreated control well. The culture plate was placed on a platform with the irradiated well positioned above a pinhole in the bearing surface (Fig. 2). The laser fiber was positioned in the center of the well at a distance of about 1.5 mm from the surface of the well bottom, allowing the laser spot to cover $∼0.8\;{\mathrm{mm}}^{2}$ of the monolayer. The fiber tip pointed in a right angle toward the bottom of the well and was positioned manually with the help of a micromanipulator (Narishige, Tokyo, Japan). Underneath the pinhole, we positioned a mirror at a 45-deg angle to divert the laser beam in a 90-deg angle, accomplishing a one-way passage of the photons through the irradiated surface. The reflected laser beam was then projected onto a screen that allowed us to assess the shape and homogeneity of the laser spot online. Cells were irradiated for 120 s with our laser pulse amplitude modulation strategy (Fig. 1) that would induce a sinusoid of 1 kHz using an LPR of 50 kHz.

The laser parameters used in our studies are shown in Table 1. After irradiation, the cells in the irradiated group as well as the controls were supplied with 100  μl of fresh culture medium and the plate was incubated at 37°C with 5% ${\mathrm{CO}}_{2}$ for the following steps of the experiment.

**Table 1:** Laser parameters used in our experiments regarding average power, energy per pulse, average radiant exposure, and average power density.

Average power (mW)	Energy per pulse (μJ)	Average radiant exposure ($J/{\mathrm{cm}}^{2}$)	Average power density ($W/{\mathrm{cm}}^{2}$)
177	3.5	2652	22.1
199	4	2988	24.9
223	4.5	3348	27.9
250	5	3756	31.3
281	5.6	4212	35.1
315	6.3	4728	39.4
354	7	5316	44.3
397	8	5952	49.6
500	10	7500	62.5

### 2.3 Fluorescence Staining and Microscopy

To assess the cytotoxicity margins of laser irradiation, we performed fluorescence staining using an Apoptotic/Necrotic/Healthy cells detection kit (Promokine, Heidelberg, Germany). The kit uses fluorescein isothiocyanate (FITC)-labeled Annexin-V, Ethidium homodimer III, and Hoechst 33342 as fluorescence markers. Membrane-permeable, minor groove-binding deoxyribose nucleic acid (DNA) stain Hoechst 33342 is used for blue-fluorescent (λabs/λem=350/461  nm) staining of the entire cell population.

**Fig. 3:**
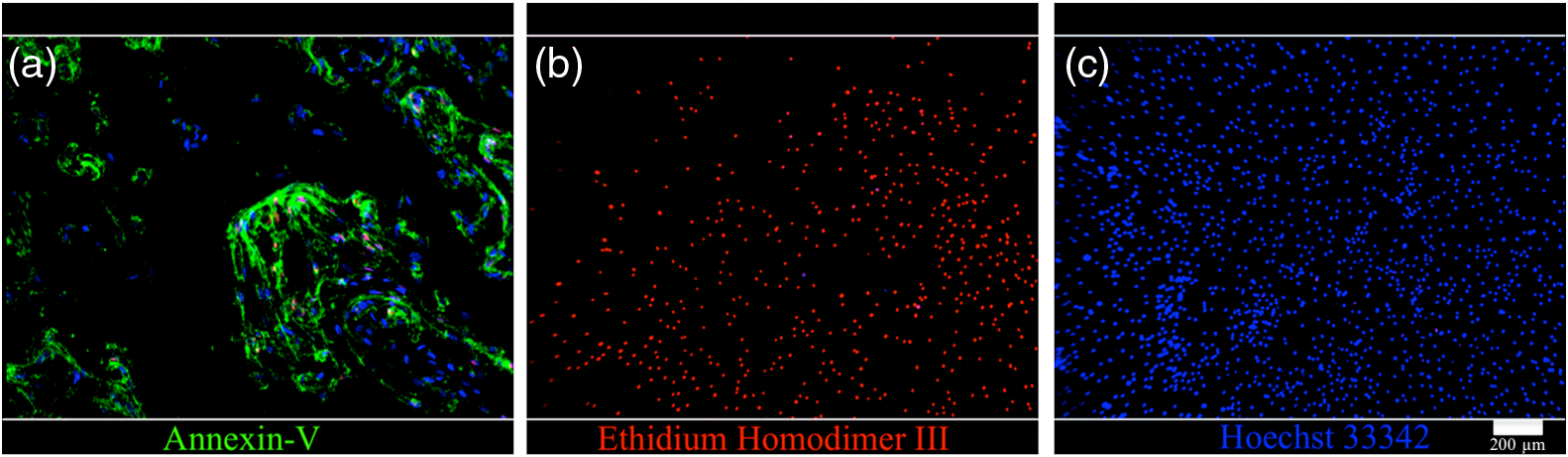
(a) A positive control for apoptosis was established using staurosporine. Apoptotic cells are stained green with FITC-Annexin-V binding to the outer membrane leaflet of apoptotic cells. (b) A positive control for detection of necrotic cells was achieved using a black filter that was placed under the glass bottom of the well being irradiated. Nuclei of necrotic cells are stained red with Ethidium Homodimer III. (c) Hoechst 33342 as a membrane-permeable probe stains the nuclei of the entire cell population. However, only healthy cells are stained blue. The scale bar represents 200  μm.

Ethidium homodimer III has a high affinity to DNA staining of the nuclei of necrotic cells red (λabs/λem=528/617  nm), but cannot enter into healthy cells. Annexin-V is a phospholipid protein with a high affinity to phosphatidylserine, which is present on the outer membrane layer during apoptosis. Apoptotic cells are consequently stained green by FITC-labeled Annexin-V (FITC; λabs/λem=492/514  nm) (Fig. 3).

After irradiation with different laser powers, the cells were washed with a binding buffer. The staining solution was prepared by adding 5  μl of the FITC-Annexin-V, 5  μl of Ethidium Homodimer III, and 5  μl of Hoechst 33342 to the binding buffer. The cells were then covered with the staining solution and incubated for 15 min at room temperature and protected from light. Following another washing step, the samples were analyzed using a fluorescence microscope (Olympus BX61, Olympus Deutschland GmbH, Hamburg, Germany).

### 2.4 Cytotoxicity Assays

#### 2.4.1 Lactate dehydrogenase assay

Lactate dehydrogenase (LDH) is an enzyme located in the cytosol of many different cell types. A loss of plasma membrane integrity during cell death leads to a release of LDH into the extracellular space. Thus, measuring the LDH activity in the culture medium can be used as a marker for cell death. With the LDH assay, we chose an enzymatic approach to assess the possible cytotoxicity of the laser treatment in every sample. In addition, although the fluorescence staining method required several washing steps associated with the loss of nonadherent cells, the LDH assay provided information about cell lysis in the entire investigated well.

After irradiation, cells were cultured for 24 h at 37°C with 5% ${\mathrm{CO}}_{2}$. LDH activity in the culture media was determined using an LDH Cytotoxicity Assay Kit (Pierce LDH Cytotoxicity Assay Kit; Fisher Scientific, Schwerte, Germany) correcting for spontaneous LDH release and intrinsic serum-LDH activity in the culture medium.

We compared every irradiated sample with the corresponding untreated control using a paired t-test. Taking into account multiple comparisons, we used the Bonferroni correction method.

In addition, we performed a baseline correction, i.e., subtracting the calculated cytotoxicity in the untreated control group from the cytotoxicity of the irradiated group.

#### 2.4.2 Water-soluble tetrazolium salt-1 assay

We chose to perform this assay to assess the potential cytostatic effects induced through laser irradiation within the wells.

Following the laser treatment, 10  μl of water-soluble tetrazolium salt (WST)-1 solution was added to 100  μl of culture medium in every well. Cells were incubated at 37°C with 5% ${\mathrm{CO}}_{2}$ for 2 h. The conversion of WST-1 to formazan by metabolically active cells was measured using an automated microplate reader (Tecan Infinite 200 Pro; Tecan, Männedorf, Germany) at a wavelength of 450 nm and a reference wavelength of 620 nm.

The results of the WST-1 assay were presented as a percentage of the control value obtained in untreated cells.

### 2.5 *RT*^2^ Profiler™ PCR Array

The effects of laser irradiation in cell cultures were assessed by quantitative polymerase chain reaction (qPCR)-array analysis for the expression of 84 genes related to stress and toxicity pathways using a real-time $RT^{2}$ Profiler™ PCR Array: Human Stress and Toxicity Finder™ (Qiagen, Hilden, Germany; Ref. PAHS-003Z).

The array included five housekeeping genes (ACTB, B2M, GAPDH, HPRT1, and PRLP0) as well as controls concerning the efficiency of the reverse transcription, the efficiency of the polymerase chain reaction itself, and the detection of genomic DNA contamination.

The cells were cultured, incubated, and treated as described above.

#### 2.5.1 RNA isolation

After an incubation period of 2 h post laser irradiation, we performed total ribonucleic acid (RNA) extraction using the QiaShredder™ Column system and the RNeasy^®^ Micro Kit (Qiagen, Hilden, Germany), including a genomic DNA elimination step. Isolated RNA was eluted in 14  μl of RNase-free water and quantified using the Nanodrop ND-1000 spectrophotometer (NanoDrop Technologies Inc, Wilmington, Delaware, USA).

#### 2.5.2 First-strand cDNA synthesis

We achieved first-strand complementary DNA (cDNA) synthesis using the $RT^{2}$ First Strand Kit (Qiagen, Hilden). Therefore, 600 ng of total RNA were reverse transcribed in a final volume of 20  μl containing another genomic DNA elimination step. Reverse transcription was performed at 42°C for 15 min and stopped by heating the probe at 95°C for 5 min. The cDNA was diluted to 111  μl final volume by adding RNase-free water and stored at −20°C until use.

#### 2.5.3 PCR array

The PCR components mix was attained by mixing the cDNA with the RT2 SYBR green/ ROX qPCR master mix (SABiosciences, Frederick, Maryland, USA) and RNase-free water according to the manufacturer’s instructions. Each well of the 96-well ${\mathrm{RT}}^{2}$ Profiler array plate (Qiagen, Hilden, Germany; Ref. PAHS-003Z) containing predispensed specific primer sets was loaded with 25  μl of the PCR components mix.

The qPCR reaction was performed for 10 min at 95°C for the activation of the Hot Start DNA Taq Polymerase followed by 40 cycles of 15 s at 95°C and 1 min at 60°C for fluorescence data collection with an ABI Step One Plus instrument (Applied Biosystems^®^ Life Technologies, Darmstadt, Germany).

We analyzed data using the ΔΔCt-method. The ΔCt for each pathway focused gene in the array plate was calculated by subtracting the average Ct value of the housekeeping genes from the gene of interest’s Ct value. Ct values >35 cycles were considered nondetectable. The ΔΔCt value was calculated according to the difference between the ΔCt of the laser irradiated group and one of the untreated control groups. The fold change was calculated by 2(ΔΔCt) representing the expression level of the sample from the irradiated group in relation to the untreated control group. A fold change of ±1.5 was chosen as a cutoff value to determine an expression level relevant for further investigation. Statistical analysis was performed using the Student’s t-test with p-values <0.05 leading to the rejection of the null hypothesis. We did not perform any correction for multiple comparisons since the qPCR array was used as a screening method.^10^

## 3 Results

### 3.1 Fluorescence Staining

**Fig. 4:**
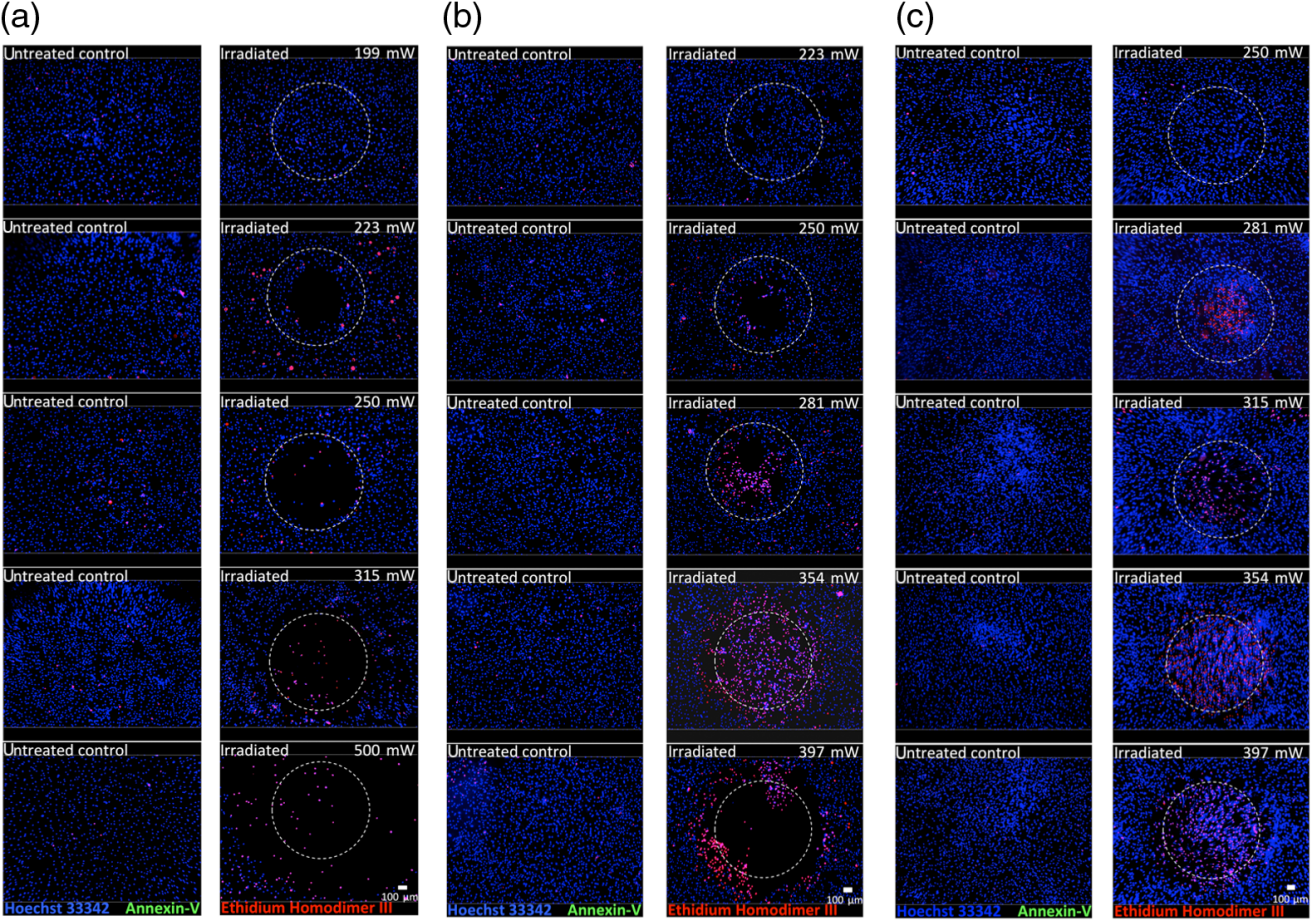
Fluorescence staining with the Apoptotic/Necrotic/Healthy cells detection assay (Promokine, Germany) after irradiation of the (a) fibroblasts (NHDF), (b) chondrocytes (HCH), and (c) osteoblasts (HOB) at different average laser powers (right side of each column) and the corresponding untreated control (left side of each column). The irradiated region is representatively marked as a white dotted circle in the right column. Dead cells are stained red (Ethidium Homodimer III), apoptotic cells are stained green (Annexin-V), and vital cells are stained blue (Hoechst 33342). The scale bar represents 100  μm.

After irradiation of human fibroblasts with laser powers up to 199 mW ($2988\;{J/\mathrm{cm}}^{2}$), no significant increase in the induction of necrosis or apoptosis in comparison to the nonirradiated group could be observed. Following irradiation with 223 mW ($3348\;{J/\mathrm{cm}}^{2}$), discrete circular areas of necrotic cells could be detected beginning in the center of the irradiated spot and increasing with increasing laser power [Fig. 4(a)].

Similar behavior is demonstrated in the HCH and HOB. However, for the chondrocytes, a first upper margin could be determined between 223 mW ($3348\;{J/\mathrm{cm}}^{2}$) and 250 mW ($3756\;{J/\mathrm{cm}}^{2}$) [Fig. 4(b)] and for the osteoblasts, the first cytotoxic effects could be observed at 285 mW [Fig. 4(c)].

### 3.2 LDH Assay

**Fig. 5:**
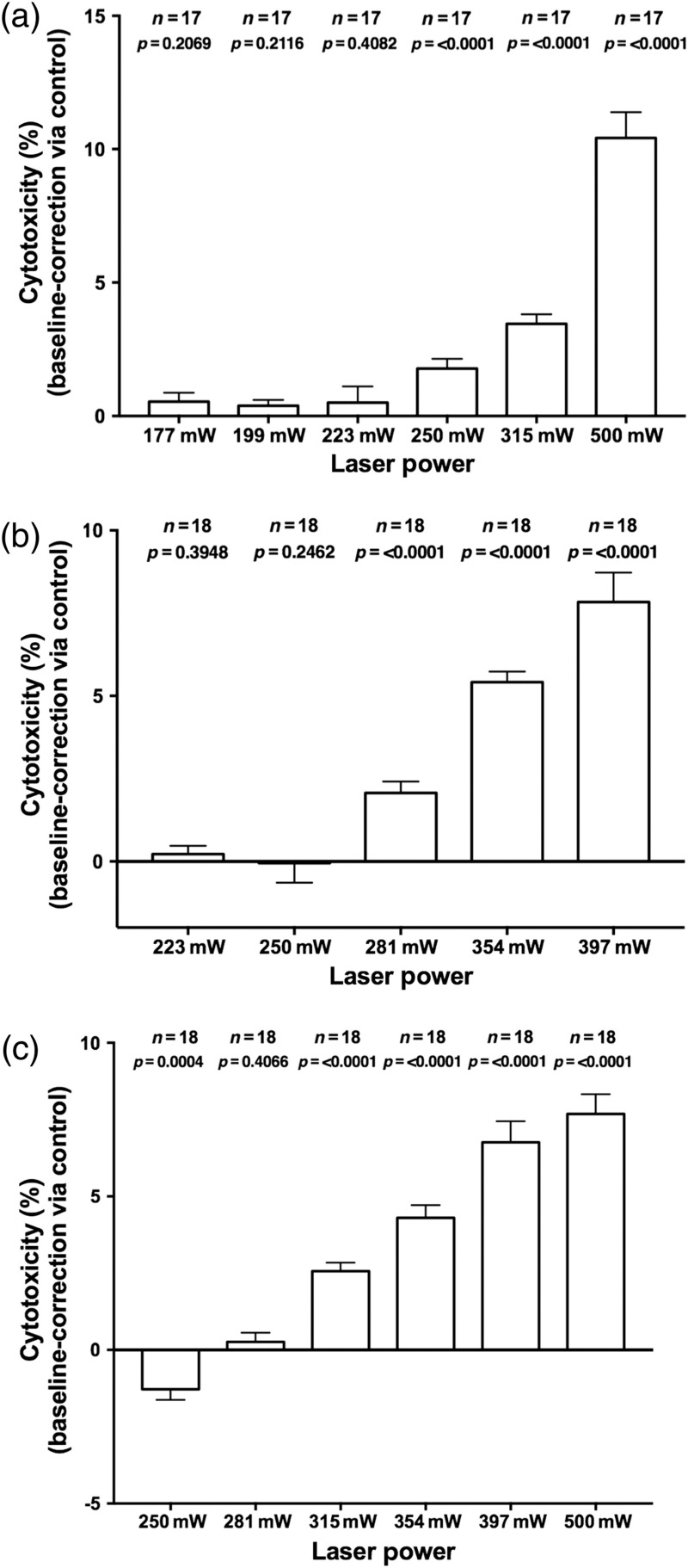
Results of the LDH assay displayed as the cytotoxicity in % after irradiation of the (a) fibroblasts (NHDF), (b) chondrocytes (HCH), and (c) osteoblasts (HOB) at different average laser powers. Error bars represent the standard error of the mean (SEM). Adjusted level of significance after Bonferroni correction: (a) and (c) p*=0.008 and (b) p*=0.01.

After irradiation of the fibroblasts with laser powers up to 223 mW ($3348\;{J/\mathrm{cm}}^{2}$), no significant cytotoxicity in comparison to the nonirradiated wells could be observed. Following the irradiation with 250 mW ($3756\;{J/\mathrm{cm}}^{2}$), a significant difference from the control group (p<0.0001), with a mean laser-irradiation-associated cytotoxicity of *circa* (ca.) 1.8% could be detected. With growing laser power, a significant increase in the cytotoxic response in the irradiated group with a mean of ca. 3.5% at 315 mW and ca. 10.4% at 500 mW [Fig. 5(a)] could be observed.

The chondrocytes demonstrated no significant changes regarding the LDH release after irradiation with 223 mW (p=0.3948) and 250 mW (p=0.2462). However, at 281 mW ($4212{\;J/\mathrm{cm}}^{2}$), a significant cytotoxicity of ca. 2% could be detected (p<0.0001) that increased with increasing laser power [Fig. 5(b)].

The irradiated osteoblasts demonstrated a significantly higher LDH release after application of 315 mW (p<0.0001) laser power in comparison to the nonirradiated controls, increasing as well with increasing laser power.

Interestingly, after the irradiation with a laser power of 250 mW ($3756\;{J/\mathrm{cm}}^{2}$), we found a significant lower LDH release in the irradiated culture (−1.28%; p=0.0004) when compared to the nonirradiated controls [Fig. 5(c)].

### 3.3 WST-1 Assay

**Fig. 6:**
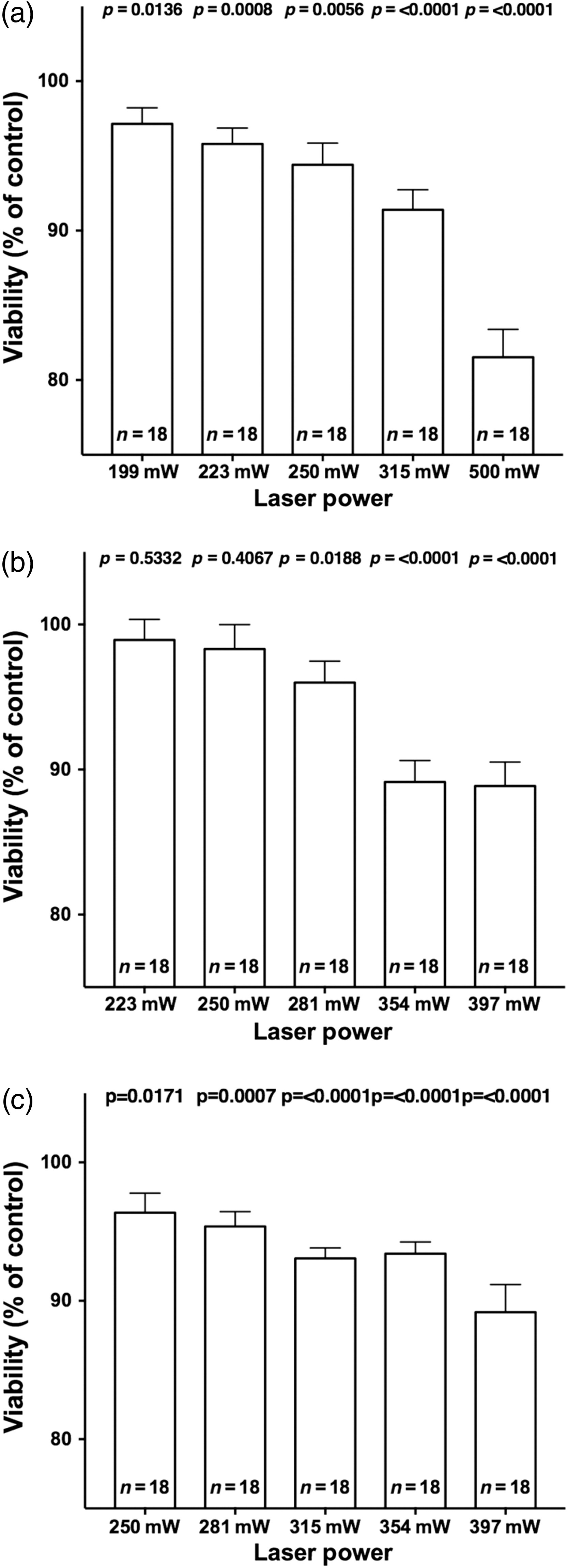
Results of the WST-1 assay displayed as the viability in % of an untreated control group after irradiation of the (a) fibroblasts (NHDF), (b) chondrocytes (HCH), and (c) osteoblasts (HOB) at different laser powers. Error bars represent the SEM. Adjusted level of significance after Bonferroni correction: p*=0.01.

After the irradiation of the fibroblasts with 199 mW ($2988\;{J/\mathrm{cm}}^{2}$) laser power, which was below our predetermined threshold for cytotoxic effects, no significant decrease in viability of the treated group in comparison to the untreated group could be detected. Following laser irradiation with a power of 223 mW ($3348\;{J/\mathrm{cm}}^{2}$), we observed a significant decrease in cell viability of ca. 4.2%, and after 250 mW ($3756\;{J/\mathrm{cm}}^{2}$) the decrease in cell viability was ca. 5.6%. After irradiation with 315 mW ($4728\;{J/\mathrm{cm}}^{2}$), the decrease in cell viability was ca. 8.7% and after 500 mW, the cell viability was ca. 18.5%. According to our prior observations, we noticed a trend for a decline in cell viability with increasing laser power [Fig. 6(a)]. This trend was also found in the chondrocytes and osteoblasts seeded wells.

The irradiation of the chondrocytes with 354 mW induced a significant decrease in viability of ca. 10.9% (p<0.0001) and the irradiation with 397 mW caused a viability loss of ca. 11.1% (p<0.0001). After irradiation of the chondrocytes with laser powers of up to 281 mW ($4212\;{J/\mathrm{cm}}^{2}$), we did not find a significant decline in viability [Fig. 6(b)].

In the wells with HOB, we noticed a first significant loss of viability after the irradiation with 281 mW ($4212\;{J/\mathrm{cm}}^{2}$), compared to the untreated control, of ca. 4.6% (p=0.0007). After the irradiation with 250 mW ($3756\;{J/\mathrm{cm}}^{2}$), no significant loss of metabolic activity of the osteoblasts could be detected [Fig. 6(c)].

### 3.4 qPCR Analysis

To develop a better understanding about the cellular processes playing a role in the changes observed after irradiation, we performed qPCR analysis for the expression of 84 key player genes known to be involved in cytotoxicity and stress response. We, therefore, irradiated cells, as described above, with laser powers below and above the cytotoxic threshold determined through our experiments and performed qPCR analysis after irradiation.

#### 3.4.1 Normal human dermal fibroblasts

**Fig. 7:**
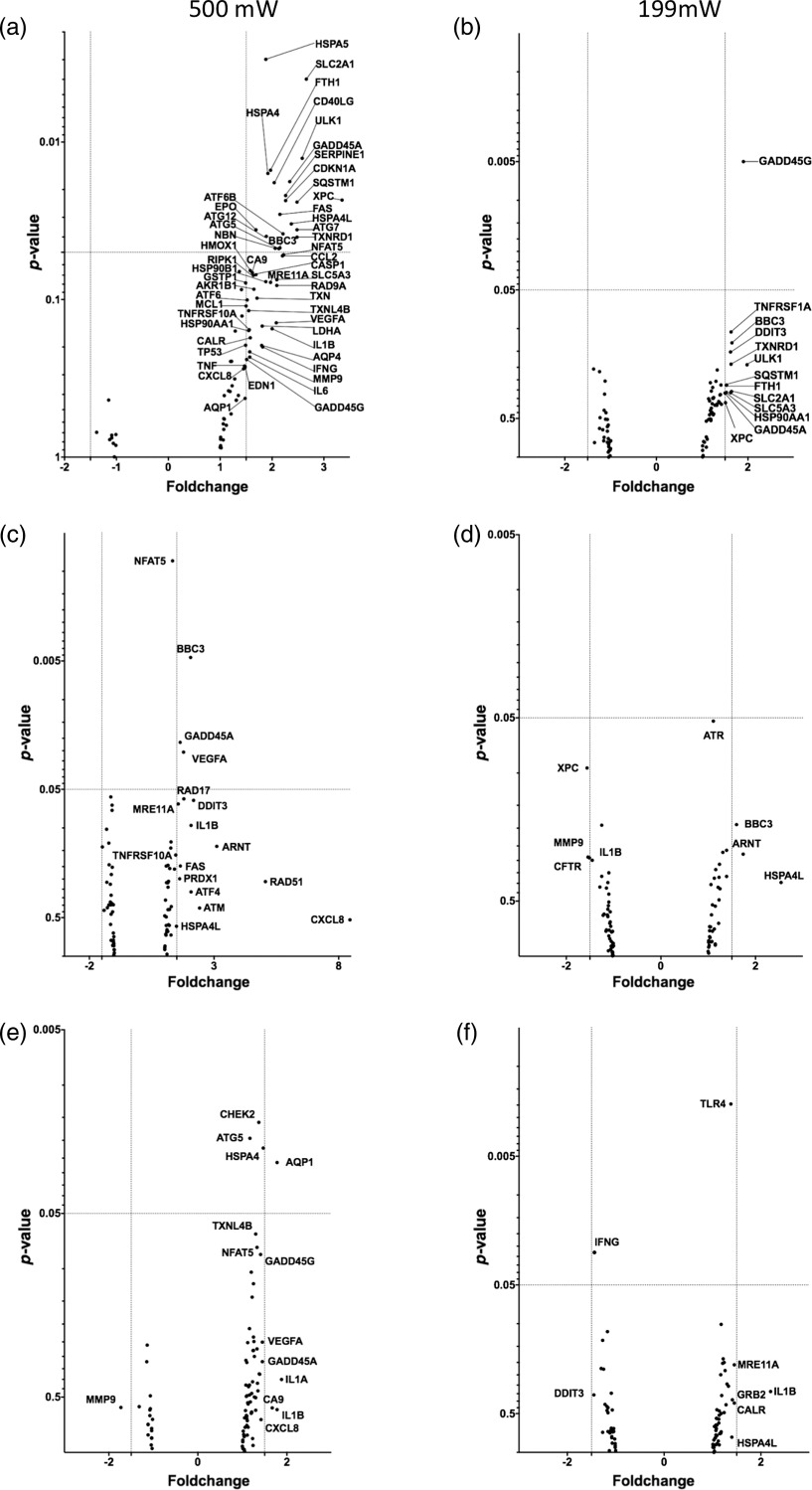
Results of the qPCR analysis displayed as volcano plots after irradiation of the (a) and (b) fibroblasts (NHDF), (c) and (d) chondrocytes (HCH), and (e) and (f) osteoblasts (HOB) with a laser power of 500 mW, which is above the cytotoxic threshold [(a), (c), and (e)] and at a laser power of 199 mW, which is below the cytotoxic threshold [(b), (d), and (f)].

After irradiation with 500 mW, we found 21 of the 84 genes significantly upregulated exceeding the fold change cutoff of 1.5. The upregulated genes derived from different pathways, such as DNA-damage response (GADD45A, XPC, NBN, and CDKN1A), oxidative stress (FTH1, SQSTM1, and TXNRD1), heat shock response (HSPA4, ATF6B, and BBC3), inflammatory response (CD40LG), hypoxia (SERPINE1 and EPO), and autophagy (ULK1, FAS, ATG7, ATG5, and ATG12). A significant relative downregulation of genes in this panel could not be observed [Fig. 7(a)].

Interestingly, following irradiation with a laser power of 199 mW ($2988\;{J/\mathrm{cm}}^{2}$), which was below the predetermined cytotoxic threshold, we found only one of the 84 genes significantly upregulated: GADD45G. A significant downregulation could not be identified [Fig. 7(b)].

#### 3.4.2 Human chondrocytes

The irradiation of chondrocytes with a laser power of 500 mW led to a significant upregulation of genes from the heat shock protein (HSP) response and unfolded protein response (BBC3), DNA-damage response (GADD45A), and hypoxia signaling-pathway (VEGFA) with a fold change >1.5. A significant downregulation of genes could not be demonstrated [Fig. 7(c)].

Following the irradiation with a laser power of 199 mW ($2988\;{J/\mathrm{cm}}^{2}$), we could find neither a significant upregulation nor downregulation of the genes in the panel [Fig. 7(d)].

#### 3.4.3 Human osteoblasts

The irradiation of the HOB with a laser power of 500 mW led to an upregulation of genes associated with osmotic stress response (AQP1), as well as HSP response/unfolded protein response (HSPA4), autophagy (ATG5), cell cycle arrest, and DNA-repair pathway (CHEK2).

Only AQP1 displayed a fold change of 1.78 while HSPA4, ATG5, and CHEK2 demonstrated fold change values below 1.5. A significant downregulation was not observed [Fig. 7(e)].

After the irradiation of the osteoblasts with a laser power of 199 mW ($2988\;{J/\mathrm{cm}}^{2}$), we found an upregulation of TLR4 with a fold change of 1.38 and a downregulation of IFNG with a fold change of −1.44, both genes referring to inflammatory response pathways [Fig. 7(f)].

## 4 Discussion

We developed an experimental setup that enabled us to define the first *in vitro* biocompatibility margins for our optoacoustic stimulation within different cell types that can be found in the peripheral hearing organ.

The fluorescence staining, the LDH assay, and the WST-1 assay could confirm a safe application of our stimulation strategy with 532-nm laser pulses *in vitro* with laser powers of up to 199 mW ($2988\;{J/\mathrm{cm}}^{2}$), suggesting the first biocompatibility margin for our stimulation parameters to be 200 to 223 mW ($3348\;{J/\mathrm{cm}}^{2}$) in our experimental setup. As expected, we found a trend of an increase in cytotoxicity with rising laser powers in all three cell types with different thresholds among them.

Although first cytotoxic effects could be detected in the fibroblasts after irradiation with an average laser power of 223 mW ($3348\;{J/\mathrm{cm}}^{2}$), the chondrocytes showed similar effects after irradiation with 250 mW ($3756\;{J/\mathrm{cm}}^{2}$) and the osteoblasts with 281 mW ($4212\;{J/\mathrm{cm}}^{2}$). These observations imply a difference in vulnerability of the different cell types to the irradiation with the parameters as described above.

There are many studies proposing specific cellular molecules as the key photoacceptors at different wavelengths. Van Breugel and Bär^11^ reported several absorption peaks in human fibroblasts around 420, 445, 470, 560, 630, 690, and 730 nm, suggesting several cellular molecules serving as photoacceptors in the visible spectrum. Karu^12^ discussed different primary mechanisms of light effects, proposing the terminal respiratory chain oxidase (cytochrome c oxidase) as the main photoacceptor molecule for red-to-near-infrared radiation, such as flavoproteins in the violet-to-blue spectral field. Furthermore, it was suggested that the different oxidation states of all cytochrome c oxidase have different absorption spectra and that the photoacceptor, after its electronic excitation, can be affected by changes in its redox properties leading to an acceleration of electron transfer.^13^^,^^14^ The biochemical activity could be induced by transient heating of the absorbing chromophore.^15^

Studies addressing the effects of light–tissue interaction on a molecular level are mainly in the optical window from 600 to 1400 nm, but there is only poor information about the effects of green laser light in fibroblasts, chondrocytes, and osteoblasts. Kassák et al. reported that irradiation of Chinese hamster ovarian cells with a wavelength of 532 nm and 30 mW, corresponding to an average radiant exposure of $1146\;{J/\mathrm{cm}}^{2}$, led to a significant increase in the mitochondrial transmembrane potential. The observations were explained with the occurrence of protoporphyrin IX as the key photoacceptor at 532 nm being part of the heme molecule, for example, in cytochrome c.^16^^,^^17^ Disregarding the differences in the laser modulation modes between our experiments and the setup described by Kassák et al., effects on the mitochondrial function and absorption of the laser light by parts of the respiratory chain should be taken into consideration. The primary underlying effects for the different sensitivities for laser irradiation between the cell types were not addressed further in this study. Our observations could be explained through varying concentrations of the photoacceptor molecules between the different cell types used or by differences in the growth patterns, respectively. Considering the fact that the extent of the affected area was not only laser power- but also time-dependent, as observed in previous experiments (data not shown), the assumption of potential thermal effects as a major damage mechanism seemed the most possible in this set of experiments.

This hypothesis was also supported by the qPCR data demonstrating multiple responses from different stress and toxicity pathways after irradiation above the safety threshold, such as DNA-damage response, oxidative stress, heat shock and inflammatory response, hypoxia, and autophagy.

References for the induction of DNA-damage response pathways following laser irradiation at a wavelength of 532 nm have been reported in the human liver cell line HepG2^18^ and hamster fibroblasts.^19^ However, the comparability of our results to these studies is very limited due to the use of a 532-nm laser with picosecond pulses and differences concerning the average radiant exposure and power density (Obringer et al.^18^: average radiant exposure: $46.7\;{J/\mathrm{cm}}^{2}$ and Leavitt et al.^19^: average power density: $30\;{\mathrm{GW}/\mathrm{cm}}^{2}$).

A laser-associated induction of the heat shock response could be observed by Bowman.^20^ Human keratinocytes pretreated with the HSP-inductor Herbimycin A showed an increased viability following laser irradiation. Hence, the induction of the HSP response was interpreted as a cytoprotective mechanism.

Khan et al.^21^ observed an increased sensitivity of laser-irradiated cells pretreated with an HSP inhibitor, thus supporting the hypothesis of a cytoprotective effect by HSP upregulation. Furthermore, they found evidence for an interrelation between the endoplasmic reticulum-stress response and HSP upregulation after laser irradiation.

Lepock et al.^22^ stated that the nuclear matrix reacts as a thermolabile cell structure, creating a link to HSP- and DNA-repair pathways. The knockout of ATF-4 led to a diminished HSP activity and autophagy response, while ATF-4 overexpression resulted in a reduced laser-associated toxicity.

An increased autophagy response following irradiation with 532 nm at a laser power of 2 W over an exposure time of 30 s (average radiant exposure: $477.5\;{J/\mathrm{cm}}^{2}$) was observed by Krmpot et al. using a rat glioma cell line. Similar to our findings, the extent of laser-induced cytotoxicity was laser power dependent. After irradiation with cytotoxic laser powers evidence for a laser-mediated induction of autophagy response was demonstrated. Cells treated with an autophagy inhibitor after irradiation showed an increased cytotoxicity. From this observation, they concluded that autophagy was induced as a cytoprotective response mechanism.^23^

Interestingly, the experimental setup was very similar to the one used in our presented *in vitro* study. However, a cytotoxicity of 50% was already observed after applying $477.5\;{J/\mathrm{cm}}^{2}$, while necrosis and total destruction of cell structures were found after irradiation with $1910\;{J/\mathrm{cm}}^{2}$. These different cytotoxic thresholds might be due to a higher vulnerability or altered response mechanisms to the laser irradiation in the glioma cell line used in their study.

The qPCR analysis and the induction of the multiple stress and toxicity pathways do not explain the genesis of the cytotoxic effects, but provide some important information about potential underlying effects and their interrelation.

We only found a significant upregulation of the stress response gene GADD45G following irradiation of the fibroblasts with a laser power of 199 mW ($2988\;{J/\mathrm{cm}}^{2}$), which is below the predetermined cytotoxic threshold within our experiments.

Interestingly, the degree of metabolic activity impairment 2 h after irradiation often exceeded the amount of LDH release measured after 24 h. Considering our findings that the key player in the cell cycle arrest^24^ GADD45G was upregulated, these observations could be explained as a potential induction of growth arrest after irradiation with subphototoxic laser powers. This finding is in consensus with Kim et al.^25^ who observed a protective mechanism induced by an upregulation of GADD45A after irradiation with visible red light.

In addition, an interesting side effect could be noticed in the osteoblasts after irradiation with a laser power below the predetermined cytotoxic threshold 250 mW ($3756\;{J/\mathrm{cm}}^{2}$) demonstrating a significantly lower LDH release than the nonirradiated control group.

Several studies in the field of low-level laser therapy (LLLT) report that light of different wavelengths can have stimulating effects on cell proliferation, recovery, and metabolic activity. Stein et al.^26^ demonstrated that LLLT of 632.8 nm and 10 mW for 3 s ($0.43\;{J/\mathrm{cm}}^{2}$) could significantly promote proliferation and differentiation of HOB *in vitro.* Similar observations were reported by Fujihara et al.,^27^ who found an increased proliferation rate of rat calvarial osteoblast-like cells after irradiation with 780 nm and an average radiant exposure of $3\;{J/\mathrm{cm}}^{2}$. An increased fibroblast proliferation could also be observed after irradiation with light-emitting diodes at 950, 660, and 570 nm at average radiant exposures ranging from 0.1 to $1\;{J/\mathrm{cm}}^{2}$ and the highest proliferation rate occurred after exposure to green light.^28^ Anwer et al. observed an increased proliferation and mitochondrial activity after irradiation of adipose tissue-derived stem cells with a wavelength of 532 nm and a laser power of 30 mW. These effects were mainly found after exposure times of 30 and 45 s corresponding to radiant exposures of 5 and $6.8\;{J/\mathrm{cm}}^{2}$. They explained their findings with an increasing activity of respiratory chain components serving as photoacceptors at a wavelength of 532 nm.^29^ However, comparing these results with our findings is very difficult, since the experimental setup such as cell type and irradiation modalities (wavelength and continuous wave mode application) was different from ours. In addition, the radiant exposures used in our experiments are much higher than in these studies. Nevertheless, our observations could implicate this additional positive effect of our stimulation strategy on human cells using laser powers below the cytotoxic threshold. Although biostimulatory effects were not the focus of this study, findings from the field of LLLT should be regarded as important clues for a better understanding of underlying photochemical effects and further applications.

Last but not least, our previously published *in vivo* studies in mice (Sorg et al.^8^), which we performed in parallel to the *in vitro* studies, demonstrated no significant damage of the irradiated area of the tympanic membrane at an average laser power of 50 mW corresponding to an average radiant exposure of $3000\;{J/\mathrm{cm}}^{2}$. First circular lesions could be observed after irradiation of the tympanic membrane with an average laser power of 89 mW and an average radiant exposure of $5340\;{J/\mathrm{cm}}^{2}$. Considering the differences in irradiation geometry between the *in vivo* performed by Sorg et al. and our *in vitro* studies, the average power density or the average radiant exposure is a more appropriate parameter for a comparison of the safety margins found for our stimulation strategy. In our *in vitro* studies, the upper limit could be found after treatment with a radiant exposure of $2988\;{J/\mathrm{cm}}^{2}$. These results display a good correlation between the upper thresholds for no significant cytotoxic effects in our *in vitro* as well as in the *in vivo* study by Sorg et al.

In our previously published *in vivo* studies (Sorg et al.^8^), a limiting factor for a very short distance between the fiber tip and the irradiated area was the conical shape and tilted angle of the mouse eardrum. However, we could accomplish a better fine-tuning regarding the limits for first cytotoxic effects in our *in vitro* model due to the flat surface of the cell cultures. In addition, we used three different adherent human primary cell types to mimic natural conditions as closely as possible.

The differences in the cytotoxicity thresholds observed in the cell types used in this study might be due to a variability in the distribution of photoacceptor molecules among the different cell types. Although the position of the fiber and the distance from the irradiated surface could be controlled with a higher precision than in the *in vivo* studies, minor variations concerning power density and homogeneity of the beam profile should be considered.

Additionally, our *in vitro* model represents only a monolayer culture. The tympanic membrane is, however, an epithelial structure consisting of different cell types and connective tissues organized in layers. It contains bone, cartilage, and blood vessels. Therefore, it creates a more complex absorption pattern for laser light when compared to the monolayer cell culture. However, this *in vitro* model offers the great advantage of analyzing the sensitivity for each cell type separately giving additional insight into the different sensitivity patterns of irradiated structures.

## 5 Conclusion

We successfully established an *in vitro* cell culture system for the cytotoxicity thresholds of the optoacoustic stimulation of the hearing organ. Our data suggest that the first *in vitro* biocompatibility margin for our stimulation parameters can be found between 200 and 223 mW ($3348\;{J/\mathrm{cm}}^{2}$). After irradiation with a subphototoxic laser power of 199 mW ($2988\;{J/\mathrm{cm}}^{2}$), only the qPCR analysis of the fibroblast culture revealed a significant upregulation of GADD45G. This could be a clue for cell cycle control mechanisms as a response to laser irradiation with sublethal laser powers. Further studies are necessary to analyze laser-irradiation-associated thermal and photochemical effects and define the optimal parameters for the optoacoustic stimulation.
